# Five years to 2030: reaching underserved populations is key to ending the AIDS epidemic in Europe

**DOI:** 10.2807/1560-7917.ES.2024.29.48.2400778

**Published:** 2024-11-28

**Authors:** Viviane Bremer, Anastasia Pharris

**Affiliations:** 1 Department for Infectious Disease Epidemiology, Robert Koch Institute (RKI), Berlin, Germany; 2 European Centre for Disease Prevention and Control (ECDC), Stockholm, Sweden

**Keywords:** HIV, UNAIDS targets, HIV-pre-exposure prophylaxis (PrEP), key population, migration, transgender

In 2025, the global community will approach a milestone year to measure interim progress towards the UNAIDS and the Sustainable Development Goal 3.3 targets “to end the epidemic of AIDS” by 2030 [1,2]. This 2025 milestone provides impetus to reflect on how far the European Union (EU) and European Economic Area (EEA) countries have progressed towards the elimination of AIDS in the last decade and to focus efforts for the remaining 5 years on how to accelerate progress.

The ambitions of the UNAIDS 2030 framework and the World Health Organization (WHO) European Regional Action Plans for ending AIDS and the epidemics of viral hepatitis and sexually transmitted infections 2022–2030 are clear: to end the HIV epidemic as a public health threat by ensuring that 95% of people with HIV are diagnosed, 95% of diagnosed individuals are receiving treatment, and 95% of those receiving treatment are virally suppressed. These indicators sum up so that cumulatively at least 86% of people with HIV should achieve a suppressed viral load by 2025, resulting in a decline in HIV incidence of 75% and a decline of 50% in HIV-related deaths by 2025 compared with a 2010 baseline. Furthermore, by 2025, less than 10% of people with HIV and key populations should experience stigma and discrimination and 500,000 people at high risk for HIV in the EU/EEA should access pre-exposure prophylaxis for HIV (Table) [2,3]. As we stand at the brink of 2025 and reflect on these targets, it is evident that, while significant strides have been made in the past 10 years, critical gaps remain.

**Table:** 2025 HIV milestones towards the 2030 UNAIDS and SDG 3.3 targets for the European Union and European Economic Area, current status, and progress made since 2014 [6,8]

2025 milestone	Current status EU/EEA, 2024	Progress made since 2014
500,000 people at high-risk for HIV receiving pre-exposure prophylaxis	159,800	First European clinical trial results were communicated in 2014. No EU/EEA country was providing PrEP outside of trial settings in 2014.
75% decline in new HIV infections from a 2010 baseline	-34%	-7% in 2014 compared with 2010 baseline [6].
95% of people with HIV diagnosed	92%	In 2014, 77% of people with HIV in countries able to measure this indicator^a^ were diagnosed [8].
95% of people with HIV diagnosed receiving treatment	93%	In 2014, 81% of those diagnosed in countries able to measure this indicator^a^ were receiving treatment [8].
95% of people with HIV receiving treatment virally suppressed	93%	In 2014, 89% of those receiving treatment in countries able to measure this indicator^a^ were virally suppressed [8].
86% of all people with HIV virally suppressed (cumulative indicator 95–95–95)	79%	In 2014, 55% of people with HIV in countries able to measure this indicator^a^ were virally suppressed [8].
50% reduction in HIV-related deaths from a 2010 baseline	-30%	-18% in 2014 compared with 2010 baseline [6].
Less than 10% of people with HIV and key populations should experience stigma and discrimination	> 10% on most measures of stigma and discrimination experienced by people with HIV	No comparable European measure of stigma was available until 2021.

EU/EEA: European Union/European Economic Area; PrEP: pre-exposure prophylaxis; UNAIDS: United Nations Programme on AIDS.

^a ^Only 10 EU/EEA countries (Austria, Bulgaria, Denmark, France, Germany, Luxembourg, the Netherlands, Romania, Spain, Sweden) plus the United Kingdom and Switzerland could measure this target in 2014. In those countries, these were the corresponding proportions at the time [8].

Pre-exposure prophylaxis (PrEP) has emerged as a powerful tool for HIV prevention in the last decade [4,5]. Availability of and access to PrEP have increased in many countries and by 2024 nearly 160,000 people were estimated to be on PrEP in the EU/EEA [6]. However, more progress is needed to reach the target of 500,000 people on PrEP. The adoption of PrEP in high-risk populations, especially men who have sex with men (MSM) with a higher risk for HIV acquisition, has shown promise in curbing new infections. Still, not all EU/EEA countries have implemented PrEP programmes and there are documented barriers to PrEP access particularly for some groups, including migrants [7]. PrEP policies that exclude undocumented migrants pose a barrier to access and leave people who are undocumented migrants with inadequate access to effective HIV prevention.

The estimated number of new HIV infections has decreased by 34% since the baseline year 2010 [6]. The increasing PrEP use in MSM alongside early diagnosis and treatment as prevention is believed to have contributed to this decrease, but the decrease noted to date falls well short of the 2025 target of 75%.

In many EU/EEA countries, HIV testing and treatment programmes have expanded notably in the last decade, leading to higher rates of early diagnosis and increasing numbers of individuals receiving antiretroviral therapy (ART) (Table). Since ‘Undetectable = Untransmittable’, the increased number of people with undetectable viral loads reduces transmission of HIV. When looking at what has been achieved in the 26 EU/EEA countries reporting data in 2024 to the European Centre for Disease Prevention and Control (ECDC) Dublin Declaration HIV monitoring process on the 95-95-95 targets, we find that seven countries had met the first target of 95% or more people with HIV knowing their status and another eight countries were within 5% of reaching the target [6]. A decade ago, in 2014, no EU/EEA country had diagnosed 95% of those with HIV and many countries lacked the capacity to measure this indicator at all [8]. As concerns the second target, eleven countries had met this 95% target, seven countries were within 5% of reaching it in 2024, whereas in 2014, only one EU/EEA country had reached this target and only four EU/EEA countries had policies of treatment regardless of CD4^+^ T-cell count [6,8]. Last but not least, for the third 95% target, 15 countries have reached it already and five are within 5% of reaching it in 2024, compared with no EU/EEA countries having documented achievement of this a decade ago [6,8]. Despite these very encouraging achievements, only eight of the 26 reporting EU/EEA countries have achieved the cumulative 86% target of ensuring that all people with HIV are virally suppressed and it is estimated that one in five people with HIV in the EU/EEA in 2024 is not virally suppressed. These data indicate that many countries are not on track to reach the 2025 targets.

The newly published ECDC/WHO European HIV/AIDS Surveillance report for the year 2023 shows a decrease of HIV diagnoses since 2014 in the EU/EEA. However, when cases previously tested positive are excluded, a 12% increase in cases diagnosed is noted between 2022 and 2023 [9]. Importantly, in 2023, nearly half of the reported HIV diagnoses in the EU/EEA were among migrants, most of them either originating from sub-Saharan Africa or from central and eastern Europe. When excluding cases with an unknown region of origin, the proportion of diagnoses in migrants among all reported HIV diagnoses in EU/EEA countries rose from 47% in 2014 to 56% in 2023. The number of HIV diagnoses among migrant MSM increased by 38% between 2014 and 2023, while the proportion of HIV diagnoses due to heterosexual transmission rose from 41% to 46%.

In this issue of *Eurosurveillance*, Reyes et al. analysed trends in HIV diagnoses in migrants between 2014 and 2023 [10]. Between 2014 and 2023, HIV diagnoses reported by EU/EEA countries in migrants originating from non-EU/EEA countries increased, while those originating from another EU/EEA country decreased. After a drop during the pandemic years, the number of HIV diagnoses increased sharply in 2022 for both men and women, probably due to the movement of people fleeing the war in Ukraine. In addition, reported HIV diagnoses increased in men of Caribbean or Latin American origin, especially after the COVID-19 pandemic. More than half of people with HIV who were not born in the EU/EEA were diagnosed late and approximately one-third even diagnosed with AIDS. Compared with non-migrants, male and female non-EU/EEA born migrants had 19% and 31% higher prevalence ratio for late diagnosis.

These findings are confirmed by a recently published systematic review, which demonstrates that migrants with HIV in Europe carry a higher risk for AIDS-defining conditions, treatment discontinuation, loss to follow-up and virological failure compared with the general population [11]. Migrants, particularly those from sub-Saharan Africa, eastern Europe, and regions with higher HIV prevalence, continue to experience important challenges in accessing HIV care. Barriers to healthcare are multifaceted, including legal, linguistic, and cultural obstacles, as well as fears of discrimination or deportation, especially in the context of irregular migration status [12]. Many migrants, particularly those with undocumented residence status, may not only be excluded from PrEP, but also avoid seeking healthcare due to fear of being reported to immigration authorities or due to a lack of awareness about available services. This is for example the case in Germany, where undocumented migrants lack access to regular healthcare and are more likely to avoid healthcare and remain undiagnosed and/or untreated [13,14].

Additionally, migrant populations often encounter difficulties in navigating the complex healthcare systems of EU/EEA countries, especially when services are fragmented or poorly adapted to their specific needs. The lack of culturally competent care and language mediation, often leads to delayed testing and late-stage diagnosis. This situation is further exacerbated by lower levels of HIV knowledge and health literacy among some migrant groups, which increases the risk of undiagnosed and untreated HIV. In this issue of *Eurosurveillance*, however, Roussos et al. show that despite a high overall level of late HIV diagnosis, people of non-Greek origin had less missed opportunities for HIV testing and thus a lower chance of being diagnosed late. The authors concluded that barriers to testing should be addressed in all people [15].

A third paper in this issue of *Eurosurveillance* sheds a light on another underserved population [16]. Wang et al. conducted a Europe-wide cross-sectional survey among European trans* and non-binary individuals. Self-reported HIV prevalence was high with 2.8% for trans* and 5.5% for non-binary individuals in this study population while both groups reported high prevalences of sexually transmitted infections. Despite this being a relatively high-risk group by study design, 15% of the respondents had never tested for HIV and 70% had never used PrEP. In comparison, a large survey among trans* individuals in Germany with over 3,000 participants exhibited lower self-reported prevalences of HIV (0.7%), with 31% not being sexually active. Only 20.5% of the respondents in the German study used HIV/STI counselling within the past 5 years and the proportion of participants taking PrEP was at a similarly low level than in the survey of Wang et al. [17,18]. While the real prevalence of HIV and STI among trans* individuals may be lower than in the study of Wang et al., both studies highlight a high level of unmet healthcare needs, along with the strong need for improved services in sexual health.

Despite revolutionary medical advancements in HIV treatment and prevention, stigma and discrimination remain key barriers to achieving the UNAIDS 2030 targets. Europe-wide surveys indicate that far more than > 10% of healthcare staff have observed various types of discrimination towards people with HIV in their workplaces while well above 10% of people with HIV report experiencing stigma and discrimination from friends, family and colleagues (Table). HIV-related stigma is often compounded for marginalised groups, such as migrants, and trans* individuals, who already face societal discrimination. In these populations, the fear of being labelled as ‘HIV-positive’ or ‘at risk’ can discourage individuals from seeking HIV testing or treatment, leading to late diagnoses and poor health outcomes [6].

While overall there is a positive development towards the UNAIDS targets of 2030 in the EU/EEA, some countries and individuals are being left behind. As we approach 2030, there is a need for bolder, more far-reaching and multisectoral strategies that tailor to the unique needs of underserved populations. To reach the UNAIDS 2030 targets, innovative, community-based and community-driven approaches must be prioritised, especially for migrants, trans* people, and other high-risk populations. Some of the proposed measures are listed in the Box.

Box **Actions to consider to better reach underserved populations with HIV services in the EU/EEA**

• **Policy change and legal protections:** Addressing the structural barriers that prevent underserved populations from accessing care requires changes at the policy and legislative level. Policies must ensure that migrants, transgender people, and other vulnerable groups have equal access to healthcare, including HIV services, regardless of their legal status. Protection from discrimination, both in healthcare settings and beyond, is also essential [3,19].

• **Targeted outreach and community engagement**: Effective HIV prevention, such as the roll-out of PrEP must be tailored to the specific needs of at-risk populations. Community-based organisations, peer educators, and local leaders can play a critical role in outreach efforts, particularly in migrant and trans* communities. These grassroots efforts should focus on building trust, providing culturally competent care, and ensuring that services are accessible and non-judgmental [3,20].

• **Mobile and digital health solutions**: The COVID-19 pandemic demonstrated the potential of telemedicine and digital health tools to bridge gaps in healthcare access. Mobile clinics, virtual consultations, and digital platforms for HIV testing, counselling, and treatment management can provide greater flexibility and reduce the stigma and self-stigma associated with accessing care [3].

• **Integrating HIV services into broader health and social services:** A holistic approach that integrates HIV services with other health and social services — such as sexual and reproductive health, mental health, and substance use treatment — can ensure that individuals receive comprehensive care that addresses their full range of needs [2,3].

EU/EEA: European Union/European Economic Area.

As the EU/EEA moves towards the UNAIDS 2030 targets, the path forward is clear: while much has been achieved, there is still much to be done. Migrants, trans* individuals, and other marginalised groups remain at risk of being excluded from existing HIV prevention, testing and treatment systems. To ensure that no one is left behind, it is essential that new, innovative ways are found to reach underserved populations, break down structural barriers to prevention and care, such as scaling up community engagement, increasing the use of digital tools, offering integrated services and addressing the social determinants of health that continue to fuel the HIV epidemic. Only through targeted, inclusive, and community-driven approaches can we hope to approach the 2025 milestones and the vision of ending the HIV epidemic by 2030.

## Note

Viviane Bremer is a medical epidemiologist and head of the Unit for HIV, STI and blood-borne infections at the Robert Koch-Institute in Berlin, Germany. She is head of the WHO collaborating centre for viral hepatitis and HIV.

Anastasia Pharris is a principal expert in infectious diseases and leader of the European Centre for Disease Prevention and Control (ECDC) Sustainable Development Goal Diseases group.
